# A Pilot Study for Using Fecal Immunochemical Testing to Increase Colorectal Cancer Screening in Appalachia, 2008-2009

**Published:** 2012-03-29

**Authors:** Brenda C. Kluhsman, Eugene J. Lengerich, Angela M. Spleen, Marcyann M. Bencivenga, Linda Fleisher, Suzanne M. Miller-Halegoua, Andrew Balshem, Electra D. Paskett, Paulette Schreiber, Mark B. Dignan

**Affiliations:** Assistant Professor, Department of Public Health Sciences, Pennsylvania State University College of Medicine. Dr Kluhsman also is affiliated with the Penn State Hershey Cancer Institute, Hershey, Pennsylvania; Pennsylvania State University College of Medicine, Hershey, Pennsylvania; Pennsylvania State University College of Medicine, Hershey, Pennsylvania; Pennsylvania State University College of Medicine, Hershey, Pennsylvania; Fox Chase Cancer Center, Cheltenham, Pennsylvania; Fox Chase Cancer Center, Cheltenham, Pennsylvania; Fox Chase Cancer Center, Cheltenham, Pennsylvania; Ohio State University, Columbus, Ohio; Elk Regional Health Center, St. Marys, Pennsylvania; University of Kentucky, Lexington, Kentucky

## Abstract

**Introduction:**

The Appalachian region of the United States has disproportionately high colorectal cancer (CRC) death rates and low screening rates. The purpose of this pilot study was to assess acceptability of a take-home fecal immunochemical test (FIT) and the effect of follow-up telephone counseling for increasing CRC screening in rural Appalachia.

**Methods:**

We used a prospective, single-group, multiple-site design, with centralized laboratory reports of screening adherence and baseline and 3-month questionnaires. Successive patients, aged 50 or older, at average CRC risk and due for screening were enrolled during a routine visit to 3 primary care practices in rural Appalachian Pennsylvania and received a free take-home FIT and educational brochure. Those who had not returned the test 2 weeks later were referred for telephone counseling.

**Results:**

Of 232 patients approached, 200 (86.2%) agreed to participate. Of these, 145 (72.5%) completed the FIT as recommended (adherent) and 55 (27.5%) were referred for telephone counseling (nonadherent), of whom 23 (41.8%) became adherent after 1 to 2 counseling sessions, an 11.5 percentage-point increase in screening after telephone counseling and 84% FIT adherence overall. Lack of CRC-related knowledge and perceived CRC risk were the screening barriers most highly associated with nonadherence. Although not statistically significant, the rate of conversion to screening adherence was higher among participants who received telephone counseling compared to an answering machine reminder.

**Conclusion:**

If confirmed in future randomized trials, provider-recommended take-home FIT and follow-up telephone counseling may be methods to increase CRC screening in Appalachia.

## Introduction

Colorectal cancer (CRC) is the third leading cause of cancer death in the United States (1). The largely rural Appalachian region has high CRC death rates. The average annual age-adjusted CRC death rate in northern and central Appalachia, including Pennsylvania, exceeds that of non-Appalachian United States (2). Although screening can reduce the risk of dying from CRC, barriers to screening persist in Appalachia, including high levels of poverty, low levels of education, limited access to health care providers and facilities, and geographic isolation (2). During 2006 through 2008, only 52% of adults aged 50 or older in Appalachian Pennsylvania reported having had a colonoscopy or sigmoidoscopy in the past 5 years, less than in non-Appalachian Pennsylvania and the United States overall (2). The *Healthy People 2020* CRC screening objective is 70.5% in the general population aged 50 to 75 (3). To reach this goal, people must be offered a test they will accept and use.

The fecal immunochemical test (FIT) is an evidence-based (1,4-5), cost-effective (6), and underused (7) screening method. FIT has been used in several countries for years; however, the test is less commonly used in the United States. Curry et al (8) found colonoscopy was the standard of care among physicians in Appalachian Pennsylvania and that their awareness of FIT as an evidence-based screening method was low. Even if most providers in Appalachia recommend FIT, use might remain low if other patient barriers persist. In studies with urban low-income and racial/ethnic minority residents, telephone counseling was successful in overcoming barriers and achieving significant increases in CRC screening (9,10). Thus, if FIT is expected to become a widespread screening method, patient acceptability of FIT should be examined. The objective of this study was to assess acceptability of a take-home FIT and the effect of follow-up telephone counseling for increasing CRC screening in rural Appalachia.

## Methods

### Design

We used a prospective, single-group, multiple-site design. Our primary outcomes were 1) acceptance of a provider-recommended, take-home FIT, as measured by enrollment and initial FIT adherence rates, and 2) FIT adherence rates after telephone counseling. The study was conducted in 2008 through 2009 and guided by principles of community-based participatory research. The Community Advisory Committee of the Northern Appalachia Cancer Network, an affiliate of the Appalachia Community Cancer Network (9,10), was involved in all phases of the research. The Penn State Milton S. Hershey Medical Center and Fox Chase Cancer Center institutional review boards approved the research.

### Development of the telephone counseling intervention

Using constructs from the cognitive-social health information processing model (cognitive, affective, cultural, and economic factors; health values; and coping strategies) (11), we developed a message library of telephone scripts and a problem-solving approach to tailor the counseling protocol to help participants work through their barriers to screening. We then conducted a focus group of rural Appalachian Pennsylvania community members and CRC survivors to review the counseling scripts. On the basis of their review, we modified the counseling scripts to help address the prevailing silence around cancer and CRC in Appalachian communities. We then programmed the counseling scripts into the Population RESearch Application Generation Environment (PRESAGE) computer-assisted telephone interviewing system at Fox Chase Cancer Center and trained a master's-level telephone counselor on CRC screening guidelines and tests, the counseling scripts, and the PRESAGE data management program.

### Study sample and recruitment

We recruited participants through 3 rural primary care practices in 3 geographically separated counties in Appalachian Pennsylvania with CRC death rates (26.3%, 20.3%, 20.2%) higher than both the national (16.7%) and Pennsylvania state (19.2%) averages (12). The population of the counties was primarily white (95.4%, 97.4%, 98.5%), similar to the other 49 Appalachian Pennsylvania counties and the larger northern and central Appalachian region (13). The sites were identified with assistance of our Northern Appalachia Cancer Network community advisory committee and included a hospital-based women's primary care clinic, a stand-alone federally qualified health center, and a practice affiliated with a large primary care network. Eligibility included being aged 50 or older, at average CRC risk, and not in compliance with American Cancer Society recommendations for CRC screening. Every day, practice staff reviewed the medical records of potentially study-eligible patients scheduled for a routine appointment that day. During the patient visit and after confirming the patient as asymptomatic for bowel disease, the attending physician or nurse practitioner offered the patient the opportunity to enroll in the study. After providing signed consent, participants completed a contact information form and a 43-item baseline questionnaire that assessed demographics and CRC-related knowledge, attitudes, and beliefs as potential barriers to screening. The questionnaire was adapted from a validated instrument (14) used in a randomized trial that tested a mailed fecal occult blood test (FOBT) with and without telephone reminders on screening outcomes in rural Minnesota (15). From May 1 to September 30, 2009, we approached 232 patients, of whom 8 (3.4%) refused and 5 (2.2%) were deemed ineligible, yielding 219 (94.4%) enrolled patients. Of these, we eliminated 19 (8.7%) from the analysis who did not complete the baseline questionnaire, did not report their age, or reported having been screened for CRC during the baseline questionnaire. Thus, 200 (86.2% of 232 who consented) made up the final study sample.

### Intervention

During the patient examination, participants received a take-home InSure FIT kit (Enterix, Inc, a Quest Diagnostics Company, Edison, New Jersey) at no charge and the American Cancer Society brochure *They know how to prevent colon cancer — and you can, too* (16) and were asked to complete and mail the test in a prepaid envelope to a central laboratory within 7 days. Those who remained nonadherent at 2 weeks postenrollment, as confirmed through the laboratory's online Care360 Physician Portal, were referred for telephone counseling. Their contact data and baseline questionnaire barriers scores were uploaded to the Fox Chase telephone counselor via the PRESAGE data management system.

The telephone protocol consisted of up to 10 call attempts for a single counseling session. After 10 failed attempts, the telephone counselor left a "cue to action" reminder message on the participant's answering machine. We considered those without an answering machine lost to follow-up. For patients contacted by telephone, the counselor used the message scripts and problem-solving techniques to address barriers reported on the baseline questionnaire and during counseling. The counseling was client-centered; therefore, the duration of counseling sessions was determined by each client's needs. Participants who still remained nonadherent 2 weeks after the initial counseling call received a "booster" call to reinforce the importance of screening and address remaining barriers. All study participants were mailed a 3-month follow-up questionnaire similar to the baseline questionnaire, except demographic questions were eliminated and questions were added about the counseling process (for those counseled) and intent to be screened in the next month (for those still nonadherent after counseling). The response rate for the 3-month questionnaire was low, completed by 114 (57%) of the 200 participants, of whom only 4 had received telephone counseling. We therefore excluded the 3-month data from the analysis.

### Analysis

We classified the prevalence and types of FIT screening barriers as 5 summary categories: 1) lack of knowledge and perceived risk (eg, age to begin screening, screening frequency), 2) unpleasantness (eg, “too messy”), 3) inconvenience (eg, lack of time), 4) cost (eg, high copayment, being uninsured), and 5) literacy issues (eg, test instructions too complicated). We used descriptive statistics to characterize participants and χ2 tests to determine any demographic differences between initially adherent and nonadherent participants. We also used the Fisher exact test and odds ratios (ORs) with 95% confidence intervals (CIs) to assess the association between conversion to FIT screening and brief counseling (1-4 min) and comprehensive counseling (5-10 min), compared with answering machine reminder (<1 min). Multivariate logistic regression was used to test associations between baseline barriers and initial screening nonadherence (referral to telephone counseling), estimated as ORs. All demographic variables including sex, age, education level, marital status, household income, health insurance status, health care coverage type, and study site were entered into the multivariate model, and backward selection was used to identify significant variables. Study site (*P* < .001) and health insurance status (*P* = .04) remained significant and were included in the final model. Significance was set at *P* < .05; all tests were 2-sided. We determined that a final sample size of 200 was necessary to detect a telephone counseling conversion rate (62%) estimated from another study (6). Data were analyzed using SAS version 9.2 (SAS Institute, Inc, Cary, North Carolina).

## Results

Most study participants were female, married, and low- to middle-income, and had health insurance, a high school education or less, and a mean age of 61 (range, 50-89) (Table 1).

### FIT adherence

Overall, 145 (72.5%) initially completed the FIT and 55 (27.5%) who remained nonadherent were referred for telephone counseling, of whom 30 received brief counseling, 15 received comprehensive counseling, 8 were reached by answering machine reminder only, and 2 were lost to follow-up. Among these 55, 23 completed FIT screening after telephone counseling, representing an 11.5 percentage point increase in screening and 84% (n = 168) adherence to FIT screening overall. Fourteen used FIT after brief counseling, 6 after comprehensive counseling, and 3 after answering machine reminder only. Although not significant, the likelihood of converting to screening was higher among those who had received any telephone counseling compared to answering machine reminder (OR, 1.33; 95% CI, 0.28-6.27). Among the 45 counseled, 27 who remained nonadherent 2 weeks later received a booster call, of whom 10 subsequently completed the FIT. We found no difference between the comprehensive and brief counseling groups in FIT adherence following the booster call. The number of call attempts averaged 3 (range, 1-10 calls) for the initial counseling session and 2 (range, 1-2 calls) for the booster session. Median time to completed screening after counseling was 16 days (interquartile range, 8-33.5 d).

### Barriers associated with screening nonadherence

Among those referred for counseling, the most prevalent type of screening barrier at baseline was lack of CRC-related knowledge and perceived risk (100%), followed by unpleasantness (63.6%), inconvenience (60.0%), literacy (32.7%), and cost (32.7%), even though FIT was provided at no charge. In multivariate logistic regression, 5 barriers from the baseline questionnaire were significantly associated with initial nonadherence (Table 2).

When asked on the baseline questionnaire, "Do you have any comments about colorectal cancer screening?" participants reported FIT as preferable to colonoscopy, citing reasons such as fear of adverse effects (eg, perforation of the colon) and dislike of the preparation for colonoscopy. Additionally, 66.7% of insured participants reported not knowing whether their health insurance covered CRC screening.

## Discussion

We found that a free, provider-recommended, take-home FIT was highly acceptable among average-risk, rural Appalachian, primary care patients; 94.4% who were approached initially agreed to participate and 72.5% completed the test as recommended. Our laboratory-confirmed FIT adherence rate was higher than the 40.0% to 66.6% self-reported adherence to colonoscopy or sigmoidoscopy in the past 5 years in the Appalachian regions of 6 states, including Pennsylvania (2), suggesting that FIT may be a more acceptable screening test for Appalachian residents. These findings are supported by 2 studies. A randomized Australian trial found population-based FIT screening was almost double that of FOBT (40% vs 24%) by 12-week follow-up after introduction of FIT, dueto its brush sampling technique and lack of dietary restrictions (17). A population-based Dutch study (18) found a significantly higher completion rate forFIT than FOBT (59.6% vs 46.9%). Thus, ease of obtaining the test specimen with FIT may partially account for our observed high initial FIT acceptance/adherence rates. It is also plausible that the high adherence rate may have been due in part to provider recommendation, one of the strongest known predictors of CRC screening (19,20). Nevertheless, our study is the first to our knowledge to demonstrate highacceptability and adherence to provider-recommended FIT screening in Appalachia and the United States.

Telephone counseling led to an 11.5 percentage-point increase in screening of initially nonadherent patients. Compared to a study by Dietrich et al (9) of low-income women in community and migrant centers in New York City, our screening adherence rate after counseling was lower (62.0% vs 41.8%). However, that investigation delivered an unrestricted number of calls for up to 18 months and assessed CRC screening of any type. A subsequent study of low- and moderate-income women in New York City by the same research team using 3 calls placed within 8 months found that women who received telephone counseling were 1.7 times as likely to be up to date with CRC screening at follow-up (10). Our findings more closely align with this latter study.

Although 3 of 8 of our participants reached only by answering machine reminder completed the FIT, it is unclear whether these recorded messages served as a cue to action to complete screening. Mosen and colleagues (21) found that in a large managed care population,up to 3 automated telephone calls and a reminder call (if needed) significantly improved FOBT testing by 6-month follow-up. Additional studies are needed to determine whether telephone counseling can yield a higher rate of screening than automated calls. In addition, our booster call had little effect among those counseled, suggesting that eliminating the booster call in future interventions could reduce costs otherwise incurred by the counselor's extra time and effort.

Our finding that lack of CRC-related knowledge and perceived risk was the most prevalent type of barrier to screening is supported by Vanderpool and Huang (22). Data from the Health Information National Trends Survey (23), which included a newly created "Appalachia residence" variable, showed that, compared with non-Appalachians, Appalachian residents had significantly greater perceived risk of developing cancer in the future, were significantly more likely to associate cancer with death, believe people can know they have cancer before a diagnosis, believe everything causes cancer, and avoid physician visits when having symptoms. The 5 variables we found associated with screening nonadherence (benefits of screening don't outweigh the risks; a normal test result doesn't lessen the need to worry about CRC; early detection doesn't make CRC easier to treat; screening might be physically uncomfortable; screening is too difficult to understand) are thus amenable to telephone counseling and warrant future additional research. Health care providers and counselors should address these barriers to screening and remain mindful that cultural norms, beliefs, and the social stigma of cancer that persists in Appalachia may underlie patients' reluctance to discuss screening. As a focus group participant stated, "No one talks about colorectal cancer in our community, not even in the newspaper." Future telephone counseling interventions should include scripts tailored to the target audience. Our finding that most insured participants did not know whether their health insurance covered CRC screening suggests that future telephone counseling interventions should include policy-level information, such as state mandates for insurance coverage of CRC screening.

This study had several limitations. Our sample was limited to mostly white, primary care patients scheduled for a routine visit, most of whom had health insurance. We did not collect data on race/ethnicity; however, the population of the 3 study counties was 94.2% white, similar to all 52 Appalachian Pennsylvania counties and slightly more than the entire 13-state Appalachian region (13). Future research in a more racially/ethnically diverse area of Appalachia could validate our results. We enrolled only patients due for screening; thus, the prevalence of being current with screening recommendations was zero. Our study may have biased patients toward initial screening adherence because the FIT was offered at no cost. It was not possible to determine which participants could have had the test reimbursed by their health insurance. However, our finding that most did not know whether their insurance covered any CRC screening lessens potential bias of the free FIT. The study period was too short to evaluate longer-term intervention effects, such as FIT rescreening. Finally, the response rate to our 3-month questionnaire was low. Our experience in rural communities suggests that a more personalized approach, such as a telephone interview or reminder call, might generate a higher follow-up response rate.

This study had several strengths. It was conducted in a part of Appalachia that has high CRC death rates by researchers who have extensive experience in this population. Community involvement ensured cultural and literacy appropriateness. Finally, the study used theory-based counseling delivered by telephone in the privacy and convenience of participants' homes, which otherwise might not be available to this rural population (24).

Provider-recommended FIT and follow-up telephone counseling may increase CRC screening in Appalachia. A randomized trial is needed to determine the effect of the intervention in the larger Appalachian population.

## Figures and Tables

**Table 1.** Characteristics of Primary Care Patients Recruited for FIT Screening, Appalachian Pennsylvania, 2008-2009

**Characteristic**	Study Sample, n (%) (N = 200)	FIT Completed, n (%) (n = 145)	FIT Not Completed; Offered Telephone Counseling, n (%) (n = 55)	Unadjusted *P* Value^a^	Adjusted *P* Value^b^
**Sex**					
Male	50 (25.0)	29 (20.0)	21 (38.2)	.01	.50
Female	150 (75.0)	116 (80.0)	34 (61.8)		
**Age, mean (SD), y**	61 (7.8)	61 (7.8)	61 (8.0)	.09^c^	.14
**Education**					
<High school	22 (11.1)	11 (7.6)	11 (20.8)	.03	.21
High school	93 (47.0)	72 (49.7)	21 (39.6)		
Some college	83 (41.9)	62 (42.8)	21 (39.6)		
**Marital status**					
Married	132 (66.7)	99 (68.3)	33 (62.3)	.41	.63
Divorced/separated	28 (14.1)	22 (15.2)	6 (11.3)		
Widowed	23 (11.6)	16 (11.0)	7 (13.2)		
Never married	10 (5.1)	5 (3.5)	5 (9.4)		
Living with someone	5 (2.5)	3 (2.1)	2 (3.8)		
**Annual household income, $**					
<15,000	46 (25.4)	26 (19.9)	20 (40.0)	.02	.16
15,000-74,999	116 (64.1)	91 (69.5)	25 (50.0)		
≥75,000	19 (10.5)	14 (10.70)	5 (10.0)		
**Health insurance**					
Insured	176 (88.9)	133 (92.4)	43 (79.6)	.04	.06
Uninsured	19 (9.6)	10 (6.9)	9 (16.7)		
Don't know	3 (1.5)	1 (0.7)	2 (3.7)		
**Health insurance covers CRC screening**					
Yes	52 (30.4)	39 (30.2)	13 (31.0)	.71	.81
No	5 (2.9)	3 (2.3)	2 (4.7)		
Don't know	114 (66.7)	87 (67.4)	27 (64.3)		

Abbreviations: FIT, fecal immunochemical test; SD, standard deviation.

a Calculated by using χ^2^ and Cochran Mantel Haenszel tests.

b Adjusted for location and calculated by using Cochran Mantel Haenszel test.

c Calculated for categories of age (50-64 y, 65-74 y, 75-84 y, ≥85y).

**Table 2.** Barriers Associated With FIT Nonadherence Among Primary Care Patients (n = 55) in Appalachian Pennsylvania, 2008-2009

**Barrier^a^**	Odds Ratio (95% CI)^b^
**Knowledge and perceived risk**	
CRC screening benefits do not outweigh test difficulties.	6.15 (2.37-15.99)
A normal test result does not lessen the need to worry about developing CRC.	3.00 (1.47-6.10)
Early detection does not make CRC easier to treat.	2.54 (1.03-6.30)
Screening might be physically uncomfortable.	2.05 (1.01-4.15)
**Literacy**	
The screening test is too difficult to understand.	2.75 (1.05-7.20)

Abbreviations: FIT, fecal immunochemical test; CI, confidence interval.

a More than 1 reported barrier per respondent was possible.

b Derived from barriers reported at baseline, controlling for study site and insurance status. The comparison group was patients who initially completed the FIT as recommended by their physician.

## References

[1] (2010). Cancer facts and figures 2010.

[2] Appalachia Community Cancer Network. Addressing the cancer burden in Appalachia 2009.

[3] HealthyPeople.gov: 2020 Topics and objectives.

[4] (2010). Are you 50 or older? Ever been tested for colorectal cancer? Publication no. 0343.

[5] Young GP (2009). Population-based screening for colorectal cancer: Australian research and implementation. J Gastroenterol Hepatol.

[6] Heitman SJ, Hilsden RJ, Au F, Dowden S, Manns BJ (2010). Colorectal cancer screening for average-risk North Americans: an economic evaluation. PLoS Med.

[7] Allison JE (2010). FIT: a valuable but underutilized screening test for colorectal cancer — it's time for a change. Am J Gastroenterol.

[8] Curry WJ, Lengerich EJ, Kluhsman BC, Graybill MA, Liao JZ, Schaefer EW (2011). Academic detailing to increase colorectal cancer screening by primary care practices in Appalachian Pennsylvania. BMC Health Serv Res.

[9] Dietrich AJ, Tobin JN, Cassells A, Robinson CM, Greene MA, Sox CH (2006). Telephone care management to improve cancer screening among low-income women: a randomized, controlled trial. Ann Intern Med.

[10] Dietrich AJ, Tobin JN, Cassells A, Robinson CM, Reh M, Romero KA (2007). Translation of an efficacious cancer-screening intervention to women enrolled in a Medicaid managed care organization. Ann Fam Med.

[11] Miller SM, Shoda Y, Hurley K (1996). Applying cognitive-social theory to health-protective behavior: breast self-examination in cancer screening. Psychol Bull.

[12] State cancer profiles, 2011.

[13] (2011). State and county QuickFacts.

[14] Baier M, Calonge N, Cutter G, McClatchey M, Schoentgen S, Hines S (2000). Validity of self-reported colorectal cancer screening behavior. Cancer Epidemiol Biomarkers Prev.

[15] Church TR, Yeazel MW, Jones RM, Kochevar LK, Watt GD, Mongin SJ (2004). A randomized trial of direct mailing of fecal occult blood tests to increase colorectal cancer screening. J Natl Cancer Inst.

[16] (2008). They know how to prevent colon cancer — and you can, too. Publication no. 243900-Rev.01/08.

[17] Cole SR, Young GP, Esterman A, Cadd B, Morcom J (2003). A randomised trial of the impact of new faecal haemoglobin test technologies on population participation in screening for colorectal cancer. J Med Screen.

[18] van Rossum LG, van Rijn AF, Laheij RJ, van Oijen MG, Fockens P, van Krieken HH (2008). Random comparison of guaiac and immunochemical fecal occult blood tests for colorectal cancer in a screening population. Gastroenterology.

[19] Winawer SJ (2001). A quarter century of colorectal cancer screening: progress and prospects. J Clin Oncol.

[20] Klabunde CN, Vernon SW, Nadel MR, Breen N, Seeff LC, Brown ML (2005). Barriers to colorectal cancer screening: a comparison of reports from primary care physicians and average-risk adults. Med Care.

[21] Mosen DM, Feldstein AC, Perrin N, Rosales AG, Smith DH, Liles EG (2010). Automated telephone calls improved completion of fecal occult blood testing. Med Care.

[22] Vanderpool RC, Huang B (2010). Cancer risk perceptions, beliefs, and physician avoidance in Appalachia: results from the 2008 HINTS Survey. J Health Commun.

[23] (2011). Health Information National Trends Survey (HINTS): how Americans find and use cancer information.

[24] Marcus AC, Garrett KM, Kulchak-Rahm A, Barnes D, Dortch W, Juno S (2002). Telephone counseling in psychosocial oncology: a report from the Cancer Information and Counseling Line. Patient Educ Couns.

